# Population genetic analysis of the *Plasmodium falciparum* circumsporozoite protein in two distinct ecological regions in Ghana

**DOI:** 10.1186/s12936-020-03510-3

**Published:** 2020-11-27

**Authors:** Elikplim A. Amegashie, Lucas Amenga-Etego, Courage Adobor, Peter Ogoti, Kevin Mbogo, Alfred Amambua-Ngwa, Anita Ghansah

**Affiliations:** 1 Department of Biochemistry, Jomo Kenyatta University of Agriculture and Technology, Juja, Kenya; 2 Department of Biochemistry, Cell and Molecular Biology, West African Centre for Cell Biology of Infectious Pathogens, University of Ghana, Legon, Ghana; 3 Parasitology Department, Noguchi Memorial Institute of Medical Research, University of Ghana, , Legon, Ghana; 4 Disease Control and Elimination, Medical Research Council Unit, The Gambia Unit, Bakau, The Gambia

**Keywords:** *Plasmodium falciparum* circumsporozoite protein, Genetic diversity, Selection, Within-host diversity

## Abstract

**Background:**

Extensive genetic diversity in the *Plasmodium falciparum* circumsporozoite protein (PfCSP) is a major contributing factor to the moderate efficacy of the RTS,S/AS01 vaccine. The transmission intensity and rates of recombination within and between populations influence the extent of its genetic diversity. Understanding the extent and dynamics of PfCSP genetic diversity in different transmission settings will help to interpret the results of current RTS,S efficacy and Phase IV implementation trials conducted within and between populations in malaria-endemic areas such as Ghana.

**Methods:**

*Pfcsp* sequences were retrieved from the Illumina-generated paired-end short-read sequences of 101 and 131 malaria samples from children aged 6–59 months presenting with clinical malaria at health facilities in Cape Coast (in the coastal belt) and Navrongo (Guinea savannah region), respectively, in Ghana. The sequences were mapped onto the 3D7 reference strain genome to yield high-quality genome-wide coding sequence data. Following data filtering and quality checks to remove missing data, 220 sequences were retained and analysed for the allele frequency spectrum, genetic diversity both within the host and between populations and signatures of selection. Population genetics tools were used to determine the extent and dynamics of *Pfcsp* diversity in *P. falciparum* from the two geographically distinct locations in Ghana.

**Results:**

*Pfcsp* showed extensive diversity at the two sites, with the higher transmission site, Navrongo, exhibiting higher within-host and population-level diversity. The vaccine strain C-terminal epitope of *Pfcsp* was found in only 5.9% and 45.7% of the Navrongo and Cape Coast sequences, respectively. Between 1 and 6 amino acid variations were observed in the TH2R and TH3R epitope regions of PfCSP. Tajima’s D was negatively skewed, especially for the population from Cape Coast, given the expected historical population expansion. In contrast, a positive Tajima’s D was observed for the Navrongo *P. falciparum* population, consistent with balancing selection acting on the immuno-dominant TH2R and TH3R vaccine epitopes.

**Conclusion:**

The low frequencies of the *Pfcsp* vaccine haplotype in the analysed populations indicate a need for additional molecular and immuno-epidemiological studies with broader temporal and geographic sampling in endemic populations targeted for RTS,S application. These results have implications for the efficacy of the vaccine in Ghana and will inform the choice of alleles to be included in future multivalent or chimeric vaccines.

## Background

Stagnation in the decline of malaria over the last 5 years indicates that global malaria elimination targets may not be achieved without the addition of a broadly effective vaccine to complement the panel of available malaria control tools [1]. However, it has taken over 15 years to finally license a moderately efficacious malaria vaccine for implementation due to extreme levels of antigenic diversity of most vaccine candidates, which reduces their efficacy across a broad range of evolving natural parasite populations.

Efficacy data from a Phase III clinical trial conducted across 11 sites in 7 African countries in children (aged 5 to 17 months) and infants (aged 6–12 weeks) revealed that the vaccine conferred moderate protective efficacy against clinical disease and severe malaria which waned over time [2]. The vaccine conferred only 36.3% protection against clinical malaria and 32.2% against severe malaria in children aged 5–17 months who received 3 primary doses of RTS,S with a booster in the 20th month [2]. The European Medicines Agency gave a favourable scientific opinion in 2015, indicating how the benefits of protective immunity outweigh the risk and the potentially high impact this moderate efficacy could have, given the huge disease burden [3]. Subsequently, Ghana, Kenya and Malawi were selected for pilot Phase IV implementation trials that are currently underway, carried out by the Malaria Vaccine Implementation Programme (MVIP) led by the World Health Organization (WHO) [4].

The RTS,S vaccine is a malaria subunit vaccine that is formulated from a fragment of the circumsporozoite protein (CSP) of *Plasmodium falciparum* 3D7 laboratory strain fused with the Hepatitis B surface antigen and the AS01 adjuvant [5]. For cell-mediated immunity, RTS,S includes a fragment of the central NANP-NVDP repeat polymorphic B-cell epitope region and a highly polymorphic C-terminal non-repeat epitope region of PfCSP, which covers CD4^+^ and CD8^+^ T-cell epitopes denoted as TH2R and TH3R, respectively [6]. Several studies have reported high levels of polymorphism in the T-cell epitopes within the C-terminal region of PfCSP in natural parasite populations [7–10]. Although there are variations in the immunodominant central repeat region (CRR), it was hoped that antibodies targeting a single dominant epitope based on the tetrapeptide repeat NANP would provide strain-surpassing immunity. This hope was strengthened by the findings of a molecular epidemiology study in African children that showed no evidence of naturally acquired strain-specific immunity to different variants of CSP obtained using the 454 sequencing platform [8]. In addition, initial ancillary studies on Phase II clinical trials conducted at three sites, including The Gambia, Kenya and Mozambique, revealed that the immune protection conferred by the RTS,S/AS02 vaccine was not strain-specific even after vaccination [11–14]. However, these studies were based on only a few hundred isolates and were not statistically powered to detect moderate effects, such as the strain-specific immune response of the vaccine.

Subsequently, an ancillary next-generation deep sequencing analysis of Phase III trial samples in 2015 showed that the vaccine indeed conferred partial protection against clinical malaria for strain-specific vaccine alleles (50.3%) and poor protection against mismatched strains (33.3%) [15]. Additionally, recent studies of the population structure of *Pfcsp* suggest that geographically variable levels of diversity and geographic restriction of specific subgroups may have an impact on the efficacy of *Pfcsp*-based malaria vaccines in specific geographic regions [7, 16]. In particular, extreme global genetic diversity of *Pfcsp* strains has been reported, with the 3D7 vaccine strain being found only in approximately 5.0% and 0.2% in some African and Asian countries, respectively [16].

The need to explore the extent of genetic diversity and the natural dynamics of malaria vaccine antigens in endemic areas where vaccines will be deployed is a point of focus due to the polymorphic nature of *P. falciparum* antigens [15–17]. Furthermore, evolutionary factors such as selection operating on parasites differ locally owing to varying transmission patterns, ecology and degrees of acquired immunity in humans [18]. Therefore, further characterization of the genetic diversity of immune epitopes of vaccine antigens is important, especially in regions such as Ghana, where the vaccine is undergoing the Phase IV implementation trial. This should provide a broader assessment of the extent to which the local natural diversity could impact efficacy and wider implementation.

Malaria transmission in Ghana is generally perennial but with marked seasonal effects that vary with the local ecology and overall transmission intensity [19]. For control purposes, malaria transmission across Ghana has been eco-epidemiologically classified into three main zones: a forest ecology zone, with perennial but high transmission during the rainy season (May–August and October–November); a northern/Guinea savannah zone, showing seasonal and intense malaria transmission during the rainy season (June-October) but also periods of very low transmission during the dry season; and a coastal savannah zone, with low to moderate perennial transmission and a marked seasonal effect during the rainy season [20].

The implementation trial of RTS,S vaccine is being conducted in three regions, namely, the Brong-Ahafo and Volta regions in the forest ecology zone and the Central region in the coastal belt, with varying transmission levels. Understanding the extent and drivers of diversity in these regions could also have a profound impact on improving the design of future circumsporozoite protein-based vaccines. Using paired-end short-read sequences of the *Pfcsp* in parasite populations from two geographically distinct sites in Ghana**,** within-host diversity (complexity of infection) and the extent of population-specific haplotype diversity of the c-terminal region of *Pfcsp* encompassing the TH2R and TH3R epitopes were investigated. Information on diversity most relevant to vaccine escape and cross-protection are provided. PfCSP amino acid diversity and conservation were further explored. In addition, evidence of selection on *Pfcsp* that could be driving and sustaining the observed diversity was assessed.

## Methods

### Study area

**Fig. 1:**
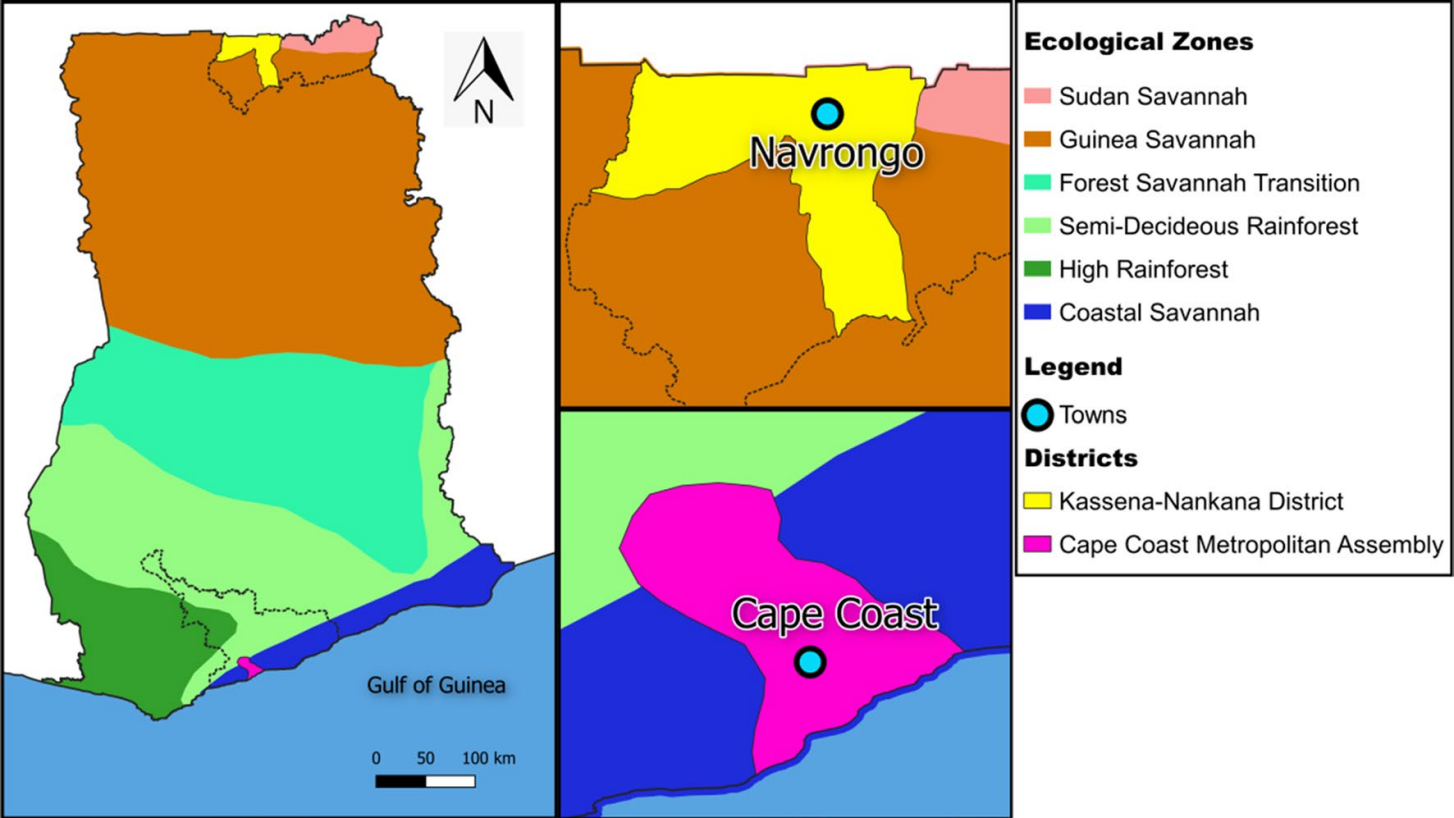
Location of Navrongo within the Kassena-Nankana Districts and Cape Coast within the Cape Coast Metropolis. The distance between Cape Coast and Navrongo is approximately 784.8 km (generated by QGIS software)

The study was conducted at two sites, the Cape Coast Metropolitan area, where Cape Coast is the main township, and the Kassena-Nankana districts (KNDs), where Navrongo is the main township (Fig. 1). Cape Coast is located in the Southern coastal savannah region, showing low to moderate perennial malaria transmission with a marked seasonal effect during the rainy season (May–August and October–November). The estimated annual entomological inoculation rate (EIR) is fewer than 50 infective bites per person per year [20]. The KNDs are located in the Upper East Region of Ghana with a Guinea savannah vegetation. Malaria is perennial in the KNDs, with high seasonal malaria transmission during the rainy season (June to October) and minimal transmission during the rest of the year, which are relatively dry months. The estimated annual EIR for the KNDs is up to 157 infective bites per person per year [21].

In Cape Coast, *P. falciparum* parasites were isolated from 101 children (aged 6–59 months) living within the municipality and presenting with clinical malaria at the Cape Coast District hospital. Samples were collected during the major rainy season (May–August) in 2013. In Navrongo, *P. falciparum* parasites were isolated from 131 children aged between 12–59 months who lived in the KNDs and presented with *P. falciparum* clinical malaria at health facilities in the KNDs in the years 2010 (January to October), 2011 (January to February) and 2013 (August to October) during both the dry and wet seasons. At both study sites, children presenting with fever, i.e., an axillary temperature ≥ 37.5° or a history of fever during the previous 24 h, were screened with malaria rapid diagnostic test (RDT). Blood smears were prepared for RDT-positive individuals and *P. falciparum* asexual parasites were identified by microscopy. Venous blood (2–5 mL) from *P. falciparum*-infected patients who gave consent was collected and archived.

### Genomic DNA extraction and sequencing

Genomic DNA was extracted using the QiaAmp DNA prep kit (Qiagen, Valencia, CA) following the manufacturer’s protocol, and the confirmation of *P. falciparum*-positive samples was performed by amplification using nested PCR with specific primers [22] (see Additional file 1). The Genomic DNA was submitted to the Wellcome Trust Sanger Institute Hinxton, UK, for whole-genome sequencing using the Illumina HiSeq platform as part of the MalariaGEN community project. Illumina sequencing libraries (200 bp insert) were aligned to the reference *P. falciparum* 3D7 genome, after which variant calling was conducted via the customized GATK pipeline. Each sample was genotyped for 797,000 polymorphic biallelic coding SNPs across the genome ensuring a minimum of 5 × paired-end coverage across each variant per sample. The dominant allele was retained in the genotype file at loci with mixed reads (reference/non-reference). The genotypes were assigned denoting the reference and non-reference nucleotides as 0 and 1, respectively. Polymorphic sites with low call rates and those in hypervariable, telomeric and repetitive sequence regions were excluded.

### Sequence acquisition and pre-processing

Genome sequences from Navrongo and Cape Coast were mined from the MalariaGEN *Plasmodium falciparum* Community (Pf3k) Project release 5.1 Database [23] in variant call format (VCF). Genetic variants on chromosome 3 were retrieved from both Navrongo and Cape Coast. In the VCF file for Cape Coast, all genotypes at each SNP position were monoallelic (monoclonal); biallelic genotypes were modelled using a custom Python script. This process was based on the approach of the MalariaGEN Pf3k Project, where loci with mixed allele calls were modelled using the read and allelic depth [24]. Briefly, to account for PCR errors, the genotypes of SNPs with a read depth < 5 were not determined. At SNP positions with a read depth ≥ 5, the samples were genotyped as heterozygous if the allelic depth of both alleles was ≥ 2. The remaining alleles were genotyped as either the homozygote reference allele or homozygote alternative/derived allele.

Data for both populations were filtered to obtain only biallelic SNPs using Bcftools v1.9 and quality checked as follows: only SNPs that passed all VCF filters were retained. Isolates with > 10% missing SNPs were excluded, followed by the removal of SNPs with > 5% missing data using PLINK v1.9 [25]. Furthermore, heterozygosity was calculated, and 8 isolates with outlier heterozygosity within the Cape Coast population were excluded. No outlier heterozygosity was observed in Navrongo. SNPs with a minor allele frequency (MAF) < 1% were removed. The remaining missing SNPs were imputed and phased using Beagle v5.1 [26]. After quality control, in the Cape Coast dataset, 2504 SNPs out of 26,156 on chromosome 3 and 92 out of 101 samples remained. However, the Navrongo dataset retained 1954 out of 43,199 SNPs on chromosome 3 and 128 out of 131 samples. *Pfcsp* was then extracted from chromosome 3 (position: 221,323–222,516) and 13 and 22 SNPs at the selected CSP loci were retained for Cape Coast and Navrongo, respectively.

### Population genetics analysis

#### Minor allele frequency distribution

Prior to the removal of rare alleles (MAF ≤ 0.01), the minor allele frequency distribution for all putative SNPs (n = 90) within *Pfcsp* for both Cape Coast (n = 35 SNPs) and Navrongo (n = 55 SNPs) *P. falciparum* isolates was determined using Plink1.9. The MAF is the frequency with which the second most common allele occurs at a given SNP position in a population.

### Within-host parasite diversity estimation and statistical analysis

The genetic diversity within the individuals was assessed by estimating Wright’s inbreeding co-efficient (*Fws*). For this analysis, the within-host diversity of *Pfcsp*, which refers to the number of different *Pfcsp* strains contained within an individual infection, was estimated. The retained variants (13 and 22 SNPs) from the 92 Cape Coast isolates and 128 Navrongo isolates were used for this analysis.

The *Fws* metric estimates the heterozygosity of parasites (H_W_) within an individual relative to the heterozygosity within a parasite population (H_S_) using the read count of alleles. *Fws* metric calculation for each sample was performed using the following equation:

$$ Fws = { 1 } - {\text{ H}}_{{\text{W}}} /{\text{H}}_{{\text{S}}} $$

where H_W_ refers to the allele frequency of each unique allele found at specific loci of the parasite sequences within the individual, and H_S_ refers to the corresponding allele frequencies of those unique alleles within the population [27, 28]. *Fws* ranges from 0 to 1; a low *Fws* value indicates low inbreeding rates within the parasite population and thus high within-host diversity relative to the population. An *Fws* threshold ≥ 0.95 indicates samples with clonal (single strain) infections, while samples with an *Fws* < 0.95 are considered highly likely to come from mixed strain infections, indicating within-host diversity. *Fws* was calculated using an R package, moimix [29]. Samples with clonal infections were used for selection analysis. The Pearson chi-squared test was used to measure the statistical significance of any differences observed in the within-host diversity estimates between the population pair. The test was performed using R software with *P* values < 0.05 considered statistically significant.

### Genetic diversity within parasite populations

The haplotype diversity (the number of two random strains within the population having different haplotypes) of *Pfcsp* in each population was determined by exploring the variants in the C-terminal region of the gene (909–1140 bp). One hundred and eighty four *Pfcsp* FASTA DNA sequences were re-constructed with the retained variants (13 SNPs) from the 92 Cape Coast isolates and 256 DNA sequences (22 SNPs) from the 128 Navrongo isolates using an in-house Python script.

The following metrics were then used to assess the diversity of the *Pfcsp* C-terminus within each parasite population using DnaSP software (version 6.10.01) [30]: number of sequences (n), number of haplotypes (h), segregating sites (S), average number of pairwise nucleotide differences (K), nucleotide diversity (π) and haplotype diversity (Hd) [31, 32].

To assess the genealogical relationships between *Pfcsp* C-terminal haplotypes found in Navrongo and Cape Coast, a network based on the method described by Templeton, Crandall, and Sing (TCS) [33, 34] was constructed using PopArt [35]. The haplotypes were denoted as 3D7, Hap 2 up to Hap 66 in the network.

In addition, amino acid haplotypes within each population were explored by translating all 440 *Pfcsp* DNA sequences (Cape Coast (184) and Navrongo (256)) into amino acid sequences and comparing them to the 3D7 reference strain (0304600.1, PlasmoDB [36]) using in-house Python scripts. The frequency of TH2R 311–327 amino acid (PSDKHIKEYLNKIQNSL) and TH3R 352–363 amino acid (NKPKDELDYAND) haplotypes in each parasite population were determined also using a customized Python script and plotted.

### Population differentiation and structure analysis

The Wright Fixation index (Fst) and principal component analysis (PCA) were used for population differentiation and structure analyses. To reduce bias in Fst analysis and PCA, SNPs (from the 2504 Cape Coast chromosome 3 retained SNPs and the 1954 Navrongo retained SNPs) with pairwise linkage disequilibrium (LD) values r^2^ > 0.5 within a window of 100 bp in the entire chromosome 3 dataset were pruned out using a step size of 10. The remaining SNPs set at chromosome 3 shared between the populations after pruning was 516, of which 10 were *Pfcsp* SNPs.

The *Pfcsp* SNPs were then used to estimate Fst and population structure. The Weir and Cockerham Fst per SNP between Cape Coast and Navrongo parasite isolates was calculated using Vcftools v0.1.5 [37] and population structure by PCA was performed using smartpca (Cambridge, MA, USA) in EIGENSOFT package v6.1.3 [38]. Principal components were computed with the number of outlier removal iterations set at 10 while maintaining other parameters. In all, 10 PCs were computed with 5 and 9 outlier samples removed from the 92 and 128 isolates from Cape Coast and Navrongo, respectively. Thus, there remained 83 samples in the Cape Coast population and 123 samples in the Navrongo population after outlier samples were removed.

### Signatures of selection

To test for SNP neutrality, the Tajima’s D statistical test [39] was performed in sliding windows with a size of 100 bp and a step size of 10 with *Pfcsp* monoclonal samples from each population using Vcftools v0.1.5. Tajima’s D test compares the average pairwise differences (pi) and the total number of segregating sites (S). Negative values indicate directional or purifying selection, while positive values indicate balancing the selection.

To detect loci likely to be under recent positive selection in the Cape Coast and Navrongo monoclonal chromosome 3 isolates, the standardized integrated haplotype score (|iHS|) was calculated for each SNP with an MAF > 0.05 on chromosome 3 (358 out of the 2504 and 608 out of the 1954 remaining SNPs from Cape Coast and Navrongo, respectively) [40]. Again, for the purpose of this analysis, the *Fws* metric was used to estimate these monoclonal chromosome 3 isolates in the retained variants within the chromosome 3 region (2504 in Cape Coast and 1954 SNPs in Navrongo).

|iHS| measures the amount of extended haplotype homozygosity (EHH) at a given SNP in the ancestral allele relative to the derived allele [40]. The reference and alternate alleles were characterized as ancestral and derived alleles, respectively. This was performed in R using the rehh package v2.0.4 [41]. Genomic regions under positive selection were identified as those with multiple SNPs showing |iHS| values > 3 and provided the focal SNPs for extended haplotype homozygosity (EHH) analysis. EHH for both the reference and alternate alleles was calculated, and bifurcation plots were generated to visualize the decay of EHH at increasing distances from the focal SNP loci [42] using rehh package v2.0.4 in R.

## Results

### Minor allele frequency distribution of *Pfcsp*

**Fig. 2:**
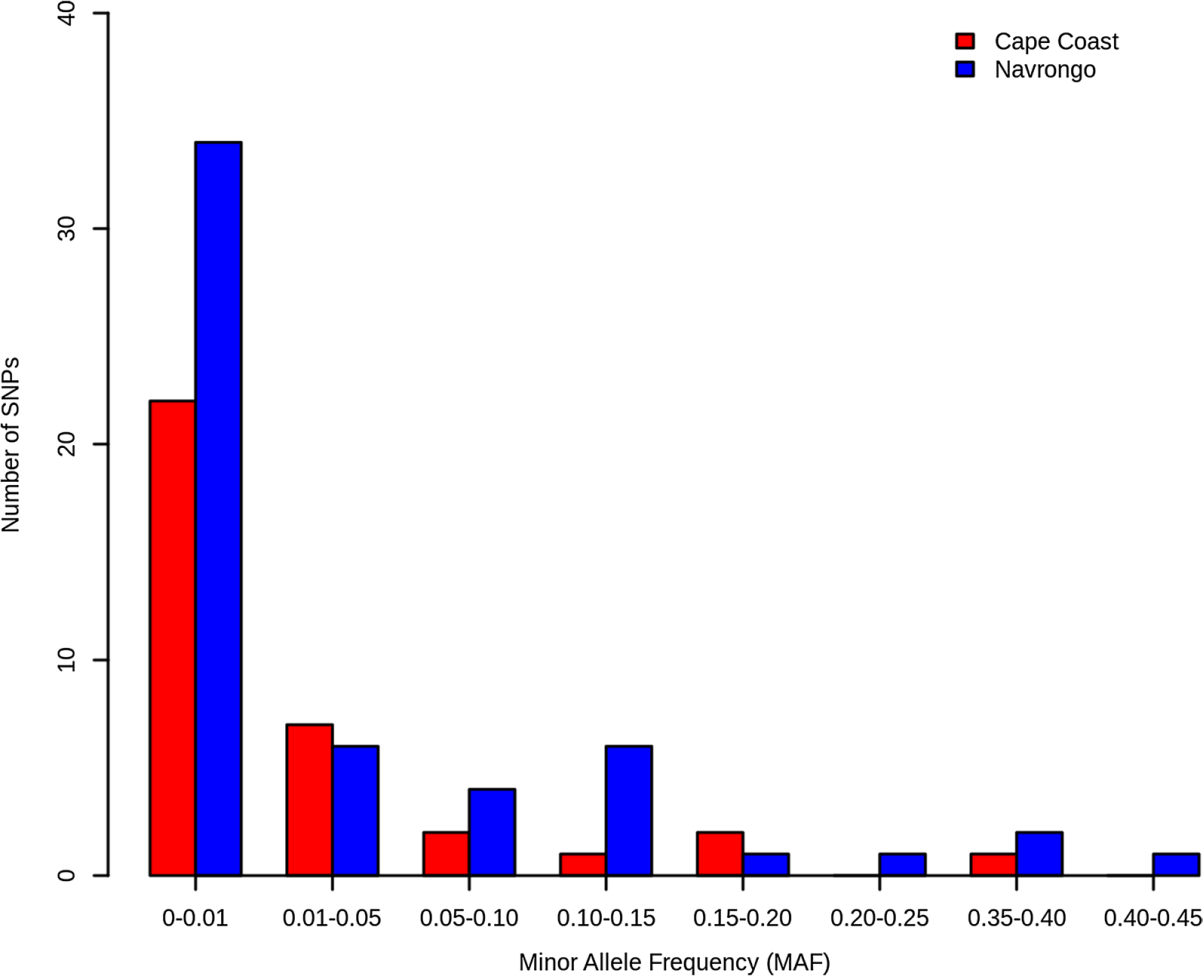
A histogram showing the minor allele frequency distribution of a total of 90 SNPs located within *Pfcsp* loci in samples from both Cape Coast (n = 35 SNPs) and Navrongo (n = 55 SNPs). The vertical axis represents the number of SNPs in each category of allele frequency, and the horizontal axis shows the SNPs set in the respective MAFs range. There were no alleles found within the [0.25–0.30] and [0.30–0.35] MAF ranges in both populations

A total of 90 SNPs within *Pfcsp* were analysed for the minor allele frequency (MAF). The *P. falciparum* population from Navrongo showed more variability in *Pfcsp* (55 SNPs) than the Cape Coast population (35 SNPs) (Fig. 2**)**. The allele frequency distribution of all putative SNPs within the *Pfcsp* loci ranged from 0.001–0.45 in Navrongo and 0.001–0.40 in Cape Coast (Fig. 2). As expected for natural *P. falciparum* populations in Africa (high transmission settings), the allele frequency spectrum was dominated by very-low-frequency alleles (MAF ≤ 0.05) in both populations. Rare alleles (MAF ≤ 0.01) were observed at frequencies of 62.9% (22/35) and 61.8% (34/55) in Cape Coast and Navrongo, respectively. A total of 20% (7/35) and 10.9% (6/55) low-frequency variants [MAF range = (0.01–0.05)] were observed in Cape Coast and Navrongo, respectively. However, the remaining alleles showed a moderate to high MAF in both populations, implying some underlying evolutionary events.

### Within-host genetic diversity of *Pfcsp*

**Fig. 3:**
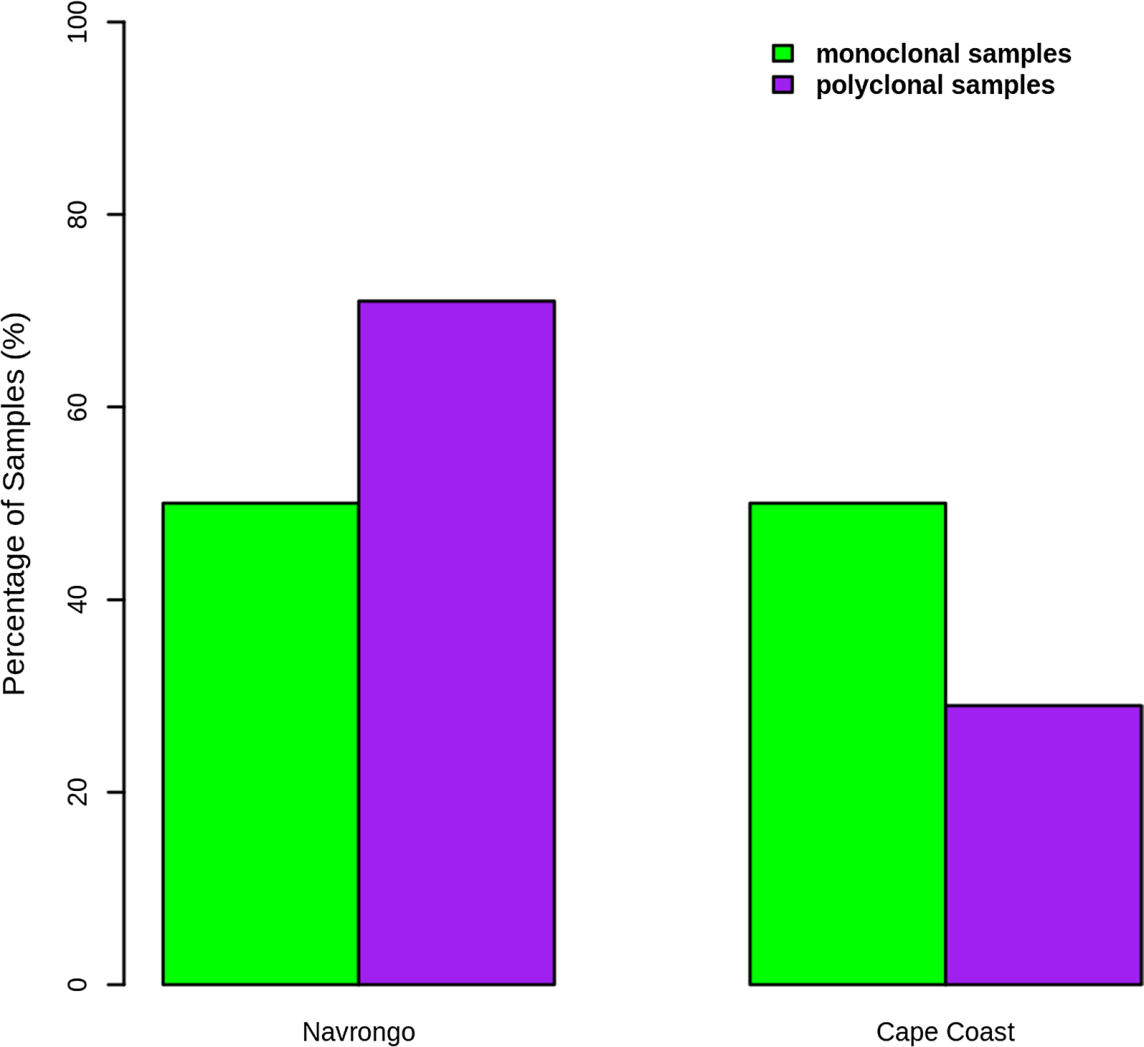
Within-host diversity. Bar plot showing the percentages of monoclonal and polyclonal samples in Navrongo and Cape Coast populations. Monoclonal samples are highlighted in green, and polyclonal samples are highlighted in purple

To assess the within-host diversity of *Pfcsp* in the population, the inbreeding coefficient (*FWS*) was investigated. Isolates with *Fws* values ≥ 0.95 were considered single strain (or monoclonal) infections, while *Fws* < 0.95 indicated diverse multigene infections. In Cape Coast, 71.7% of *Pfcsp* isolates (66/92) came from single-strain infections with high inbreeding potential, while 28.3% (26/92) came from highly diverse multistrain infections with high potential for outcrossing (Fig. 3). For *P. falciparum* infections from Navrongo, 50.8% (65/128) were monoclonal *Pfcsp* isolates, and 49.2% (63/128) harboured multiple *Pfcsp* strains **(**Fig. 3). The Navrongo *Pfcsp* isolates exhibited significantly higher within-host diversity than those from Cape Coast (*χ*^2^ = 15.382, *p* = 0.00009).

### Genetic diversity of *Pfcsp* C-terminal haplotypes

**Fig. 4:**
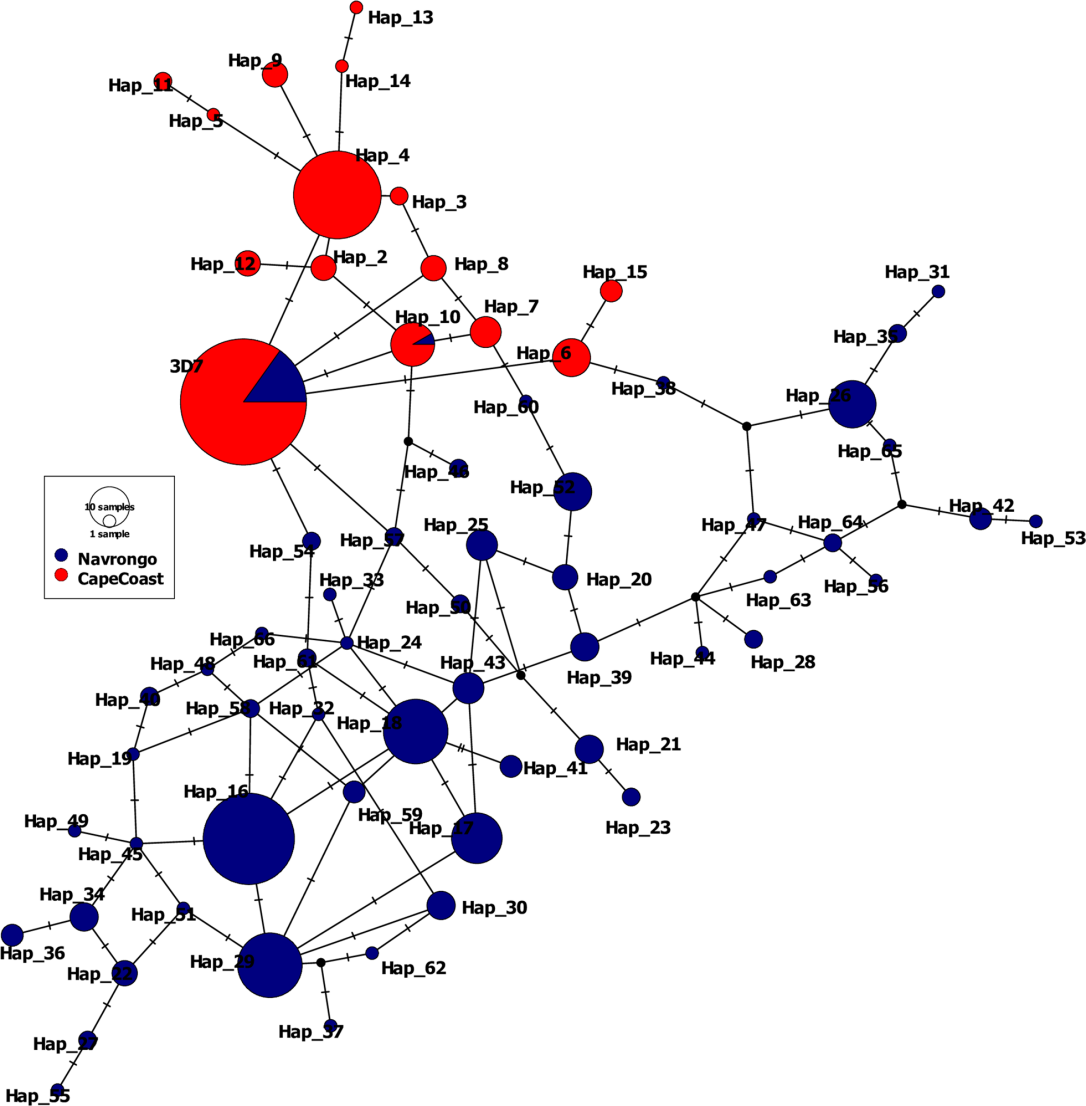
Templeton, Crandall, and Sing (TCS) network providing a summary of the diversity of *Pfcsp* haplotypes in the C-terminal region obtained from 440 DNA sequences in both Cape Coast (n = 184) and Navrongo (n = 256). Circles represent each *Pfcsp* C-terminal haplotype, and circles are scaled according to the frequency with which the haplotype was observed. Each haplotype is denoted as “Hap” with the vaccine strain 3D7 (3D7 0,304,600.1, PlasmoDB [38]) denoted as “3D7”. Haplotypes obtained from the Navrongo sequences are colour-coded blue, and haplotypes obtained from Cape Coast coded red

To assess the extent of genetic diversity and similarity within and between the two populations, the diversity in the C-terminal region of *Pfcsp* (231 bp) was investigated from a total of 440 DNA sequences from Cape Coast (n = 184) and Navrongo (n = 256) (Table 1) and summarized in a Templeton, Crandall, and Sing (TCS) network (Fig. 4).

**Table 1:** Diversity indices of the *Pfcsp* C-terminal region of samples included in the network analysis

Population	n	Calculated indices				
		h	S	K	$\pi\, \pm \,\mathrm{S}.\mathrm{D}$	Hd$\,\pm\, \mathrm{S}.\mathrm{D}$
Cape Coast	184	15	8	1.15	0.005 ± 0.0004	0.718 ± 0.026
Navrongo	256	53	16	3.76	0.016 ± 0.0007	0.925 ± 0.009

*n*  number of sequences, *h* number of unique haplotypes, *S* number of segregating sites, *K* average number of pairwise nucleotide differences, π nucleotide diversity, *Hd* haplotype diversity

In total, 66 haplotypes were observed among the 440 *Pfcsp* sequences obtained from both populations (Fig. 4). Among these haplotypes, 15 and 53 were found in the Cape Coast and Navrongo populations, respectively. The RTS,S vaccine haplotype (Pf3D7-type) and 1 nonvaccine haplotype (denoted as “Hap 10”) were found in both populations (Fig. 4). The Pf3D7-type haplotype represented only 5.9% (n = 15/256) of haplotypes in Navrongo but 45.7% (n = 84/184) in Cape Coast (see Additional file 2). Only a single sample exhibited Hap 10 from Navrongo (0.4%), but this haplotype represented 6.0% of the total haplotypes in Cape Coast (11/184 isolates) (Additional file 2). While the Pf3D7-type haplotype was the most prevalent *Pfcsp* C-terminal haplotype (45.7%) in isolates from Cape Coast, the most frequent haplotype in the Navrongo isolates was “Hap 16”, representing 20.3% (52/256) of the haplotypes detected (Additional file 2).

According to the analysed genetic diversity indices, the *Pfcsp* C-termini of the Navrongo isolates were generally more diverse than those from Cape Coast (Table 1). In summary, more nucleotide polymorphisms (K = 3.761) and segregating sites (S = 16) were observed in Navrongo than in Cape Coast (K = 1.148, S = 8). Consequently, *Pfcsp* nucleotide diversity ($\pi )$ was higher in the Navrongo isolates ($\pi =0.016 \pm$ 0.0007) than in the isolates from Cape Coast ($\pi$ = 0.005 $\pm$ 0.0004). Haplotype diversity was also higher in Navrongo (Hd = 0.925 ± 0.009) in comparison with Cape Coast (0.718 $\pm$ 0.026) parasite isolates.

### TH2R and TH3R amino acid haplotype diversity

**Fig. 5:**
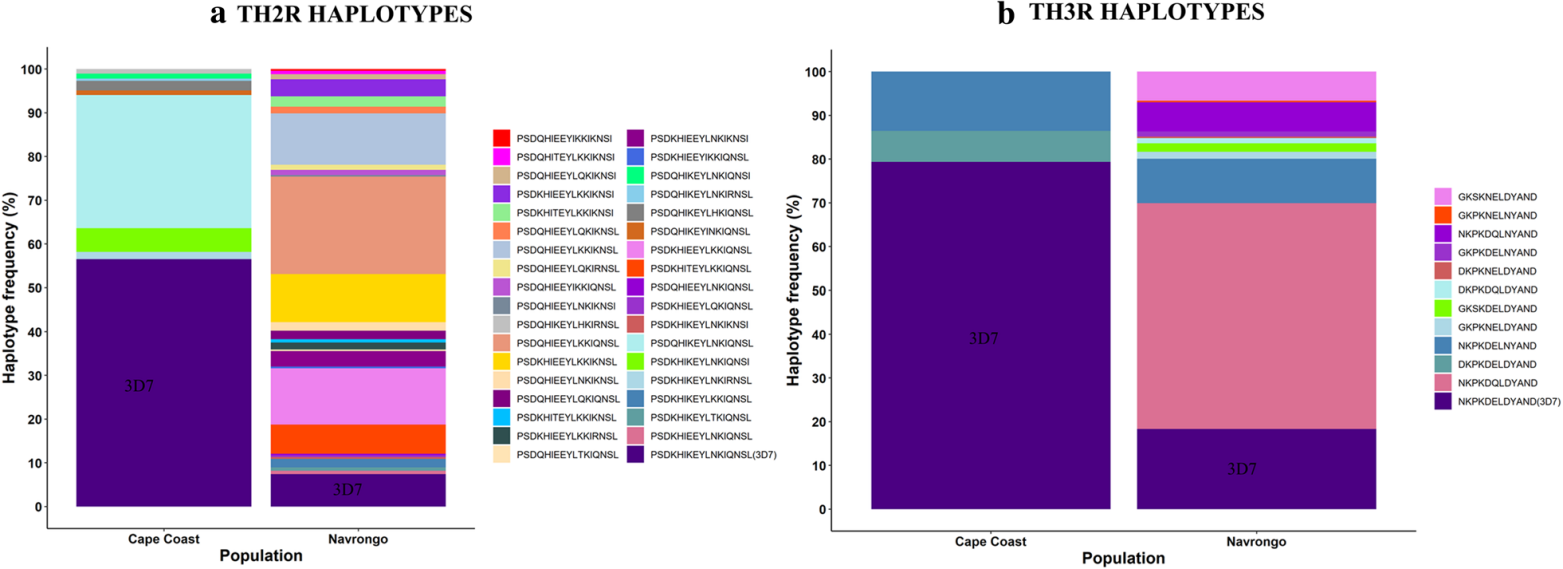
Plot **a** and** b** showing the percentage of isolates sharing specific amino acid haplotypes within the TH2R (311–327 aa) and TH3R (352–363 aa) epitope regions in both Cape Coast and Navrongo population, respectively. Coloured columns in the bar graph represent the haplotypes. The proportion of samples in each population having the 3D7 haplotype (vaccine haplotype) is represented in the first purple-coloured column from the bottom. The proportions of samples with non-vaccine haplotypes are shown in the rest of the coloured column bars

The TH2R and TH3R sites were more polymorphic in both populations than the remaining amino acid sequence in the C-terminal region of PfCSP. In general, non-synonymous mutations predominated in all the isolates in both TH2R and TH3R epitope regions, with implications for cross-protection. Among the 92 (184 amino acid haplotypes) and 128 (256 amino acid haplotypes) isolates from Cape Coast and Navrongo, there were 8 and 27 nonvaccine TH2R haplotypes, respectively (see Additional file 3). There were also 2 and 10 nonvaccine TH3R haplotypes in Cape Coast and Navrongo, respectively, with 1 nonvaccine haplotype (NKPKDELNYAND) being shared between the two populations (Additional file 3). The frequencies of the Pf3D7-type TH2R vaccine haplotype (PSDKHIKEYLNKIQNSL) were 56.5% and 7.4% in Cape Coast and Navrongo, respectively (Fig. 5a), while the frequencies were 79.3% and 18.4% for the Pf3D7-type TH3R vaccine haplotype (NKPKDELDYAND) (Fig. 5b) in the Cape Coast and Navrongo isolates, respectively. The amino acid differences observed between Pf3D7 reference (3D7 0304600.1, PlasmoDB) and the Ghanaian isolates ranged from 1 to 6 in both epitope regions (see Additional file 3).

### Population differentiation and structure of *Pfcsp*

**Fig. 6:**
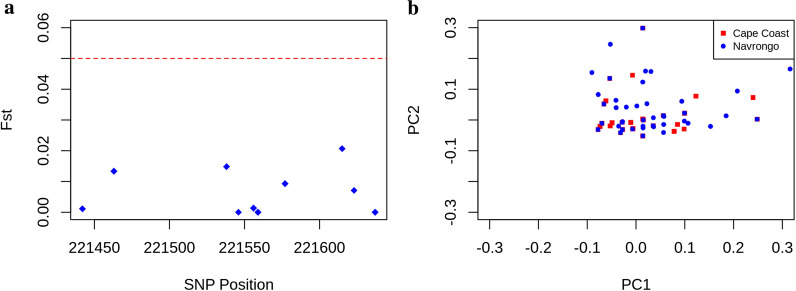
Population differentiation and structure. **a** Weir and Cockerham’s Fst calculated at SNP loci between Cape Coast (n = 92) and Navrongo (n = 128) population samples for 10 SNPs. The red line shows the borderline of 0.05 which indicates moderate population differentiation **b**. Plot of first (PC1) and second principal component (PC2) of samples in both the Cape Coast (n = 83) and Navrongo (n = 123) populations after outlier samples were removed

The overall Weir and Cockerham’s Fst between the Cape Coast and Navrongo *Pfcsp* populations was < 0.05 (Fig. 6a), which indicates minimal population differentiation due to genetic structure and suggests gene flow between the populations, despite the geographic distance between the sites. This also confirms the lack of genetic structure observed between Cape Coast and Navrongo parasite isolates through principal component analysis (Fig. 6b).

### Evidence of selection within populations

**Fig. 7:**
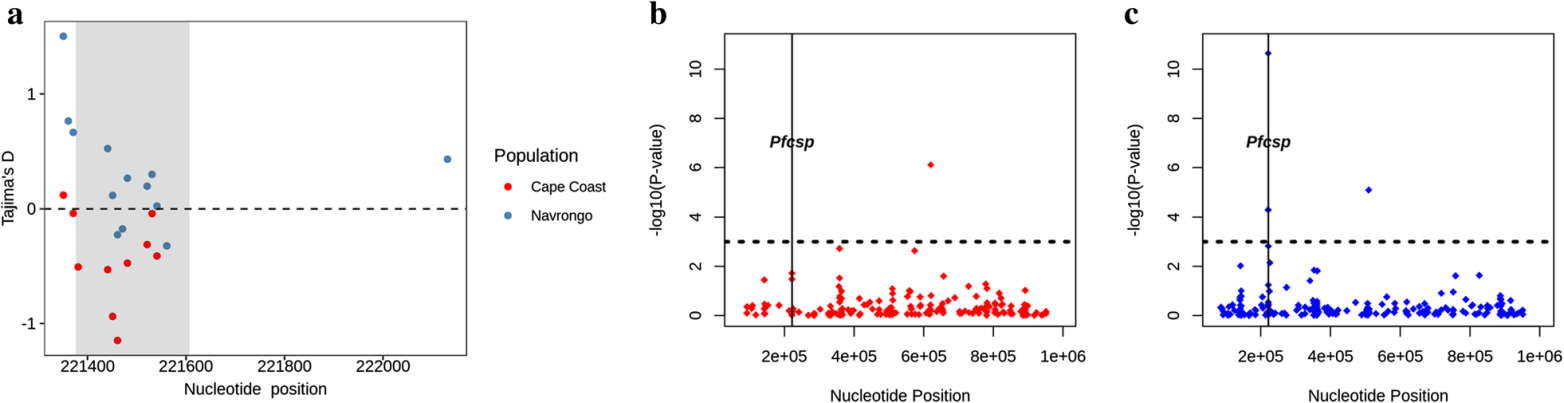
**a** Tajima’s D plot in sliding windows of 100 and a step size of 10 of 66 mnoclonal *Pfcsp* samples (Cape Coast) and 65 monoclonal samples (Navrongo). The region highlighted in grey represents SNPs located in the C-terminal region (221,422–221,583) of *Pfcsp.* Plots **b** and **c** show evidence of signatures of positive directional selection on chromosome 3 using the standardized integrated haplotype score (|iHS|) plotted as -log10 (*P* value) (for 55 and 50 *P. falciparum* chromosome 3 monoclonal isolates) in both Cape Coast **b** and Navrongo **c,** respectively. IHS was calculated for SNPs with no missing data and a minor allele frequency > 0.05. SNPs on chromosome 3 are identified in red in Cape Coast **b** and blue in Navrongo **c**. Horizontal lines indicate the threshold for high-scoring SNPs with a standardized |iHS|> 3. Vertical lines indicate the positions of *Pfcsp* in both Cape Coast and Navrongo, respectively. Evidence of positive directional selection was observed at *Pfcsp* loci in Navrongo; however, there was no evidence of selection in the *Pfcsp* loci from Cape Coast

**Fig. 8:**
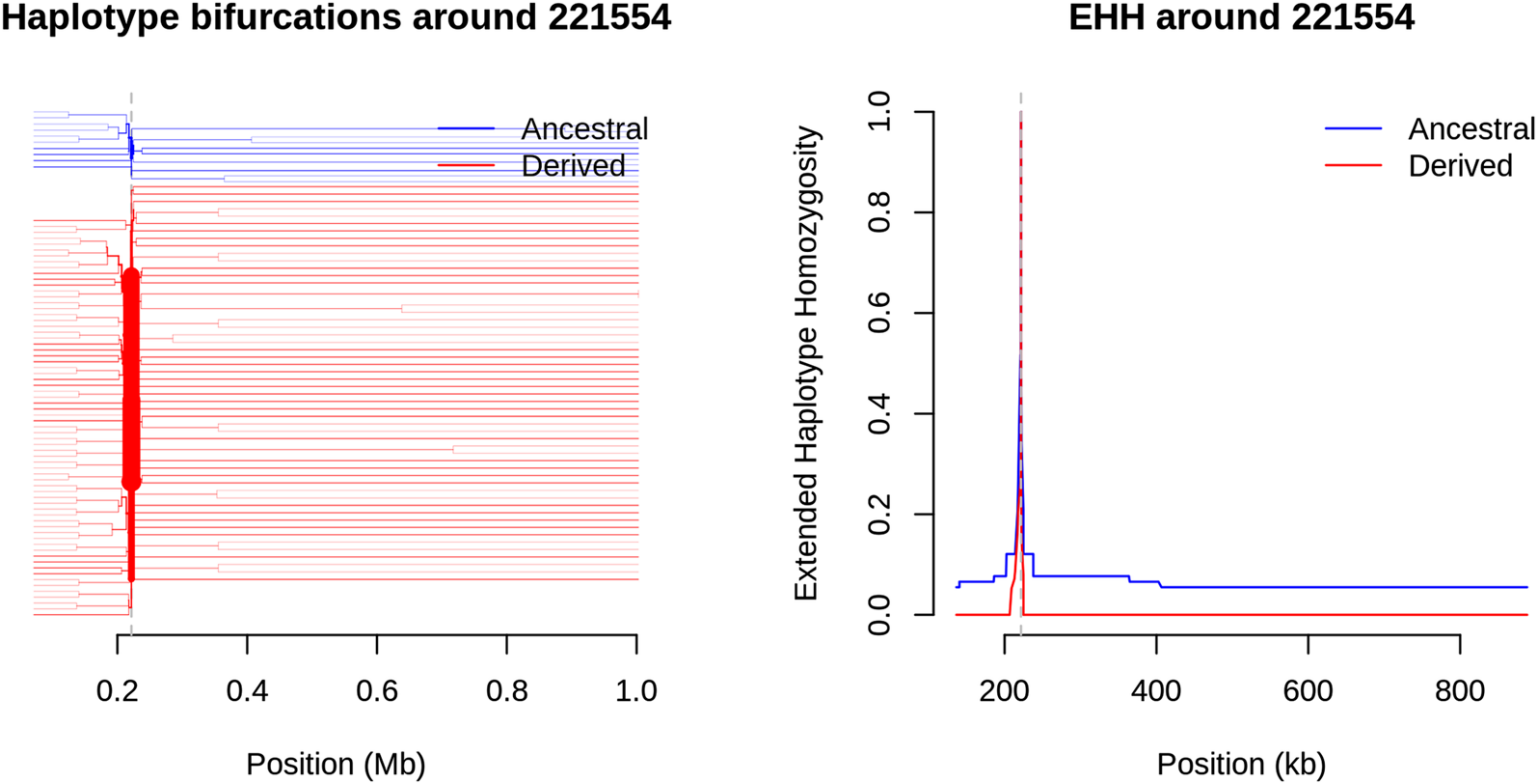
Extended Haplotype Homozygosity (EHH) and Bifurcation diagram. **a** Plots of EHH showing extended haplotypes from a focal SNP locus (221,554). **b** Bifurcation diagrams showing the breakdown of these extended haplotypes from increasing distances in Navrongo parasite population. Evidence of positive directional selection was observed at this focal SNP locus, which is found in the TH2R epitope loci of *Pfcsp*

Tajima’s D values were greater than zero in the TH2R and TH3R epitope regions of the C-terminal loci of *Pfcsp* (221,422–221,583) for the population of monoclonal *Pfcsp* isolates from Navrongo (Fig. 7a), suggesting balancing selection. However, a Tajima’s D < 0 was seen in the Cape Coast population at these loci, suggesting likely directional selection or clonal expansion in the population. Alleles at SNP locus 221,554, which is within the segment encoding the TH2R epitope, had an |iHS|> 3 in the Navrongo population, suggesting recent positive selection (Fig. 7c). The extended haplotype homozygosity revealed some extended haplotypes from the focal SNP locus 221,554 in the Navrongo population, but no long-range haplotypes extended beyond 221,554 (Fig. 8a, b).

## Discussion

The RTS,S/AS01 malaria vaccine is based only on the *Pfcsp* sequence of the *P. falciparum* 3D7 clone [16], and strain-specific immunity has been confirmed for the licensed vaccine [15]. To provide new insights into how well RTS,S/AS01 may perform if administered on a large scale in different malaria-endemic regions, it is important to assess the intra-host diversity and extent of diversity in circulating parasites from different transmission settings.

Using *Pfcsp* sequence data generated from the whole-genome sequencing of 92 and 128 clinical parasite isolates from Cape Coast in the coastal savanna region and the Kassena-Nankana districts (KNDs) in the Guinea savannah zone of the Upper East Region of Ghana, higher within-host malaria parasite diversity was observed in Navrongo, where 49.2% of infections exhibited an *Fws* < 0.95, than in Cape Coast, where only 28.3% of infections exhibited an *Fws* < 0.95. This high genetic diversity is known to occur in high-transmission areas, where infected individuals usually harbour more polyclonal infections compared to those living in low-transmission areas, where infections are often monoclonal [43]. Malaria transmission is much higher in Navrongo (EIR = 157) than in Cape Coast (EIR = 50) [20, 21]. These findings are consistent with high outcrossing potential in the parasite population in the KNDs compared to the parasite population in the coastal town of Cape Coast. This marked difference in within-host diversity is noteworthy for future region-specific vaccine intervention strategies.

High genetic diversity in the C-terminal TH2R and TH3R amino acid epitopes was observed at the two sites. Notably, the vaccine-specific Pf3D7-type haplotype in the TH2R and TH3R epitopes represented approximately 56.5 and 79.3%, respectively, of the observed haplotypes in Cape Coast and only approximately 7.4 and 18.4% of those in the Navrongo isolates. The observed variance in location-specific diversity in these epitopes, which correlates with malaria transmission intensity, is consistent with findings from previous studies [8–10, 44–46]. Such polymorphisms in T-cell epitopes have been suggested to be due to an immune evasion mechanism in response to host T-cell immune responses [10] or selection in the mosquito host during the malaria transmission cycle [44]. From 1 to 6 amino acid differences were observed within the TH2R and TH3R epitope regions at each epitope in both parasite populations, with implications for the duration of vaccine efficacy [13]. This situation is similar to the amino acid haplotype differences observed in the C-terminal region in the Zambian and DRC populations, ranging from 2 to 10 [16]. In addition, there were more amino acid substitutions in the Navrongo parasite population than in the Cape Coast parasite population, which is consistent with the lower frequency of the vaccine haplotype observed in the network analysis for the Navrongo parasite population, and this will have implications for vaccine efficacy in comparison with high-malaria-burden populations in Ghana. Another hypothesis drawn from a previous study suggests that polymorphism in the T cell epitopes could also be driven by an evolutionary response to intermolecular interactions at the surface of CSP [47].

The high degree of location-specific *Pfcsp* diversity observed in Ghana might result in differences in vaccine efficacy, potentially reducing RTS,S/AS01 vaccine effectiveness, particularly in Navrongo, where the vaccine haplotype was less prevalent. The monitoring of differential vaccine efficacy according to *Pfcsp* haplotypes during RTS,S/AS01 implementation programmes will be valuable for such high-transmission areas, where post-vaccination expansion of nonvaccine haplotypes in the population is likely to be observed, and this could lead to reduced vaccine efficacy and vaccine breakthrough infections.

The abundance of rare alleles shown in both Cape Coast and Navrongo contributes to the parasite population. Despite this high level of genetic diversity resulting from nonsynonymous nucleotide and amino acid substitutions observed, a shared gene pool remained between the two sites that resulted in a largely homogeneous parasite population. Over the sampled range of 784.4 km between the two sites, there was gene flow between the local populations of *P. falciparum* according to *Pfcsp* sequence analysis, with a pairwise index of differentiation (Fst) below 0.05. The principal component analysis further confirmed the lack of population structure or genetic isolation. Previous studies have indicated that human population mixing is likely to cause gene flow among *P. falciparum* parasites [48, 49]. Despite the ecological and epidemiological diversity between the 2 sites**,** human movement between the two sites is significant and could account for *Pfcsp* gene flow within the country, with implications for the spread of any emerging vaccine-resistant parasite. High levels of genetic recombination in the high-transmission area may explain the observed differences in haplotype diversity in Navrongo in comparison with Cape Coast [50] despite the observed gene flow between the two sites.

The negative Tajima’s D observed in the Cape Coast isolates indicates a likely population expansion of the 3D7 major haplotype in an area with moderate malaria transmission after over 15 years of enhanced nationwide malaria control interventions (chemotherapy and vector control). This result corroborates findings from Thiès, Senegal, where increased deployment of malaria control interventions resulted in an increase in the frequency of clonal strains and a decrease in the probability of multiple infections [51]. Evidence of recent positive and balancing selection was observed in the Navrongo parasite isolates. The majority of the alleles present in the C-terminal region in the Navrongo parasite population exhibited a positive Tajima’s D score and were highly polymorphic, likely due to balancing selection in response to host immune pressure on this immunogenic epitope [44, 52, 53]. Evidence of balancing selection on *Pfcsp* had been reported previously for a population from Malawi [44]. Balancing selection is common for immune targets and has been reported in other vaccine antigen candidates, such as in the domain I epitope of the Pf38 gene (found on the merozoite surface) in Papua, New Guinea, and The Gambia [54] and in the extracellular domains of AMA1, a target of allele-specific immune responses [55]. However, seasonal genetic drift among loci attributable to sampling across multiple transmission seasons in the Navrongo population may contribute to the observed balancing selection. The evidence of recent positive directional selection (iHS > 3) observed at the T-cell epitope loci in the Navrongo parasite population could be due to the addition of new and useful alleles to the already existing repertoire of alleles being maintained by balancing selection in the population [18]. However, the signature of positive selection observed in Navrongo could likely be attributed to the dominance of one allele over the others at the T-cell epitope region in the Navrongo parasite population. Considering the differences in the eco-epidemiological background and the EIRs of these two populations, the intensity of transmission at these two ecologically distinct sites could account for differences in selection signals observed [45].

The samples analysed here were nonrandomly selected from the population, and this lack of randomness may have some limitations and bias the inferences that can be drawn from *Pfcsp* and PfCSP diversity. Notably, the Navrongo and Cape Coast isolates were opportunistic samples whose sequence data were deposited into the Pf3K database at different times, leading to a geographically biased set of sequences, possibly over-representing limited genotypes from a small number of geographic foci and, in turn, under-representing large higher frequency SNPs. Furthermore, the conclusions drawn from sequences obtained from any given sequence repository are subject to change as sample sizes and geographic and temporal distributions are continually updated and expanded. Another limitation that may affect the interpretation of data is the small sample sizes analysed. Finally, the Navrongo sequences obtained from Pf3k come from different periods than the sequences from Cape Coast, which were sampled from the same periods. These limitations may prevent the samples from these two regions from being optimally comparable. There was, however, no population structure found within or between the two populations, indicating that the timespan did not affect the results obtained here. In addition, samples from Cape Coast were collected from a single district hospital, whereas the samples from Navrongo came from three health facilities. This circumstance may potentially have an impact on the results obtained, although the Cape Coast district hospital serves a wider catchment area, comparable to that of the three Navrongo sites. Despite these inherent limitations, the sequence analysis elaborated here is a powerful approach capable of elucidating local patterns in vaccine candidate genetic diversity and would be useful for monitoring the effect and efficacy of interventions. The examination of a larger sample size allowing a geographically and temporally broader analysis will further reveal the extent of the diversity of *Pfcsp* both locally and across Africa. This will help inform strategies for a wider implementation of the RTS,S vaccine.

## Conclusions

The extent of CSP polymorphism observed at the study sites likely indicates an allele-specific immune response during the pilot Phase IV implementation trials being conducted in Ghana. Similar to observations made in a study of an AMA-1 vaccine, vaccine efficacy during this trial in Ghana may be dependent on the degree of homology between the amino acid haplotypes circulating in the natural parasite populations and the 3D7 vaccine haplotype [56]. This situation might gradually result in a directional selective advantage of unmatched CSP haplotypes because the vaccine does not target them, emphasizing the need for a polyvalent malaria vaccine [57, 58].

With the ongoing Phase IV RTS,S vaccine implementation trials in Ghana, which include populations from Cape Coast and Navrongo, the findings from this study provide prior information on the extent of diversity in *Pfcsp* and the evolutionary forces driving these variations within Ghanaian natural parasite populations. These data will inform vaccine implementation outcomes and contribute to future vaccine design. These findings further emphasize the need for incorporating large-scale prevalence and population genetic analysis of vaccine candidate antigens into future malaria vaccine design to predict malaria vaccine outcomes.

## Supplementary information

**Additional file 1.** This file contains the primers used in nested PCR amplification of *Plasmodium falciparum* genomic DNA.

**Additional file 2.** This file is a Nexus file containing C-terminal *Plasmodium falciparum* circumsporozoite protein gene (*Pfcsp*) haplotype sequences that were included in the Templeton, Crandall, and Sing (TCS) network.

**Additional file 3.** TH2R and TH3R amino acid haplotype frequencies.

## Data Availability

The datasets generated during the current study are available in the MalariaGEN *Plasmodium falciparum* Community (Pf3k) Project release 5.1 (http://www.malariagen.net/projects/parasite/pf).

## Abbreviations

WHO: World Health Organization
RDT: Rapid Diagnostic Test
CSP: Circumsporozoite Protein
Pfcsp: Plasmodium falciparum Circumsporozoite Protein
NMCP: National Malaria Control Program
MVIP: Malaria Vaccine Implementation Program
AEIR: Annual Entomological Inoculation Rate
EIR: Entomological Inoculation Rate
AMA1: Apical Membrane Antigen 1
MSP: Merozoite Surface Protein
CRR: Central Repeat Region
PCR: Polymerase Chain Reaction
VCF: Variant Call Format
HBsAg: Hepatitis B Surface Antigen
SNP: Single Nucleotide Polymorphism
GATK: Genome Analysis Tool Kit
PCA: Principal Component Analysis
MAF: Minor Allele Frequency
IHS: Integrated Haplotype Score
EHH: Extended Haplotype Homozygosity
