# Supplementary material for: Population genetic analysis of the Plasmodium falciparum circumsporozoite protein in two distinct ecological regions in Ghana
Source: Malar J. 2020 Nov 27;19:437. doi: 10.1186/s12936-020-03510-3 (PMC7694917; doi:10.1186/s12936-020-03510-3)
Supplement: Supplementary file 1 — Additional file 1. This file contains the primers used in nested PCR amplification of Plasmodium falciparum genomic DNA. [file 12936_2020_3510_MOESM1_ESM.docx]

**Table 1:** **Primers used in nested PCR amplification of *Plasmodium falciparum* genomic DNA (Snounou et al., 1993).**

| **Primer** | **Sequence** |
| --- | --- |
| rPLU 6 | TTAAAATTGTTGCAGTTAAAACG |
| rPLU 5 | CCTGTTGTTGCCTTAAACTTC |
| rFAL 1 | TTAAACTGGTTTGGGAAAACCAAATATATT |
| rFAL 2 | ACACAATGAACTCAATCATGACTACCCGTC |
